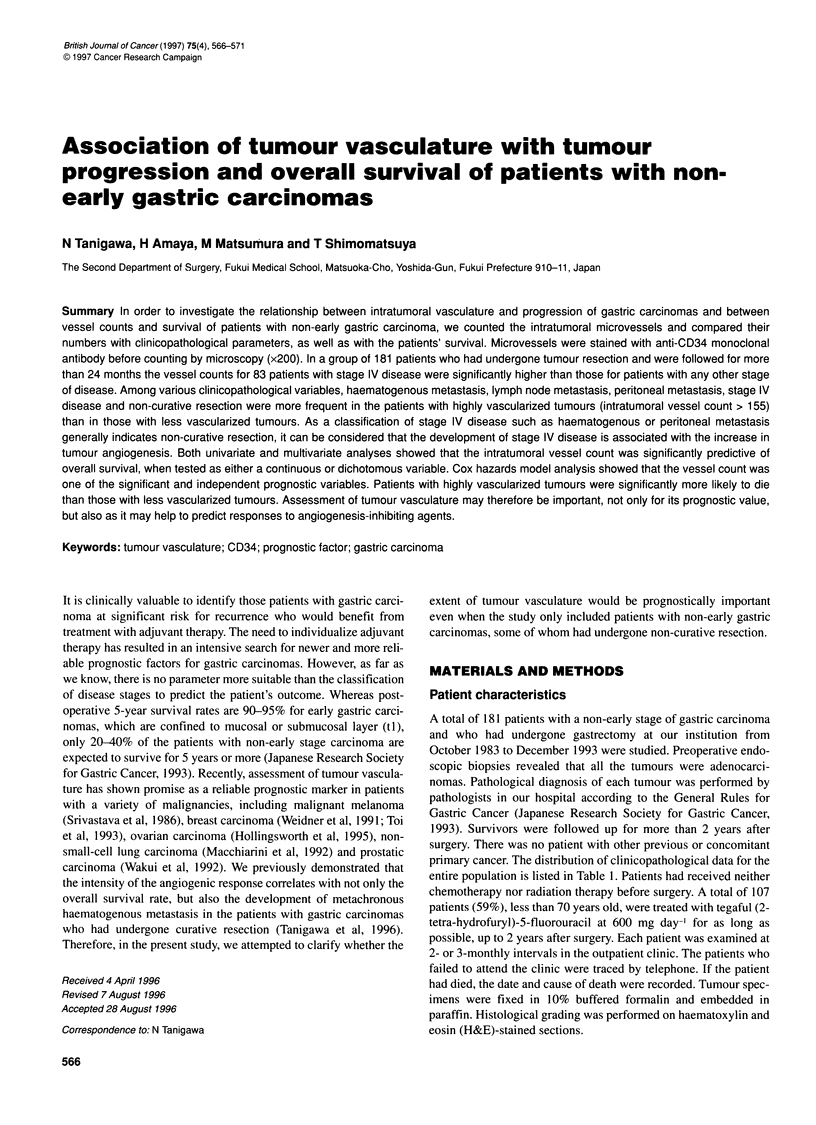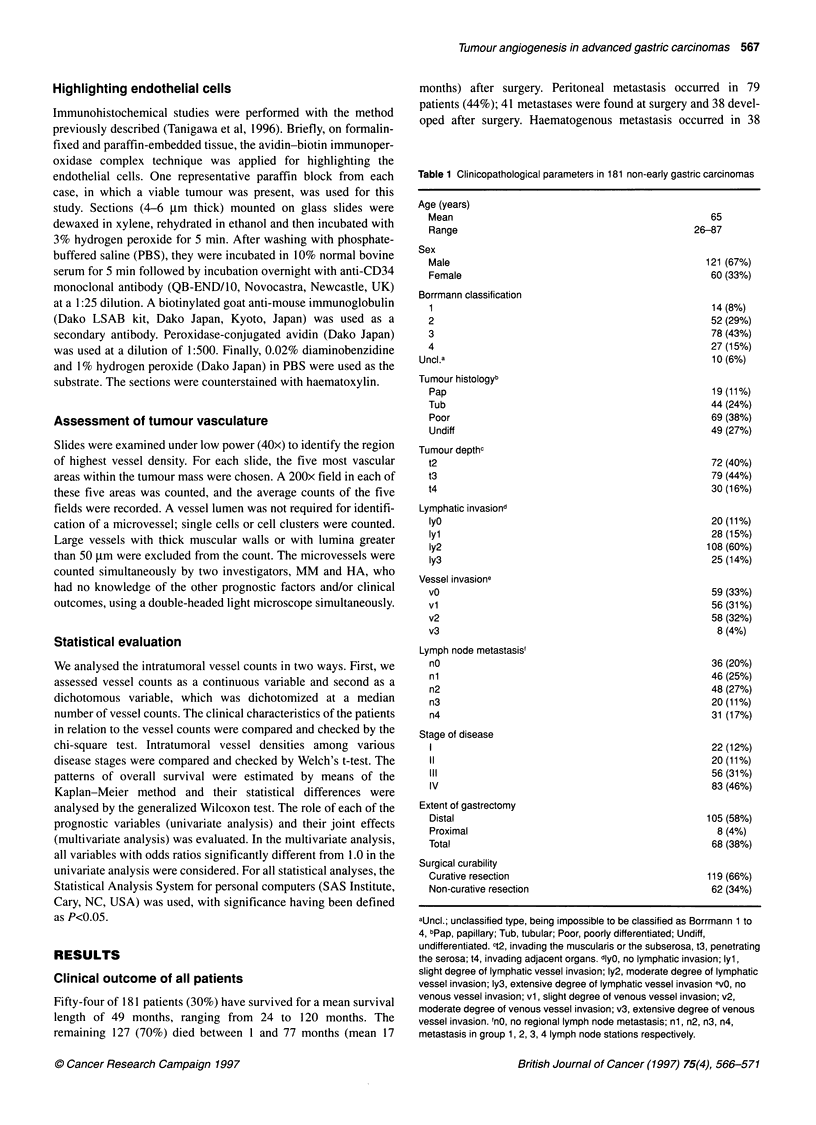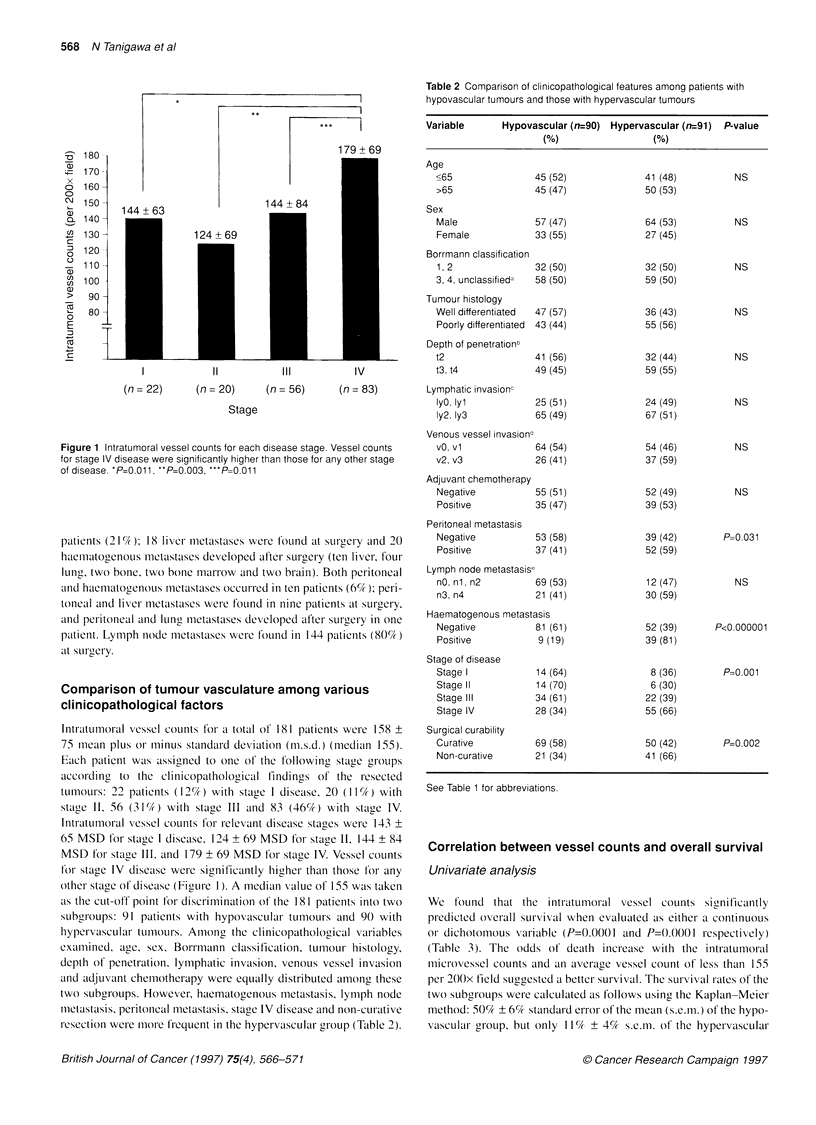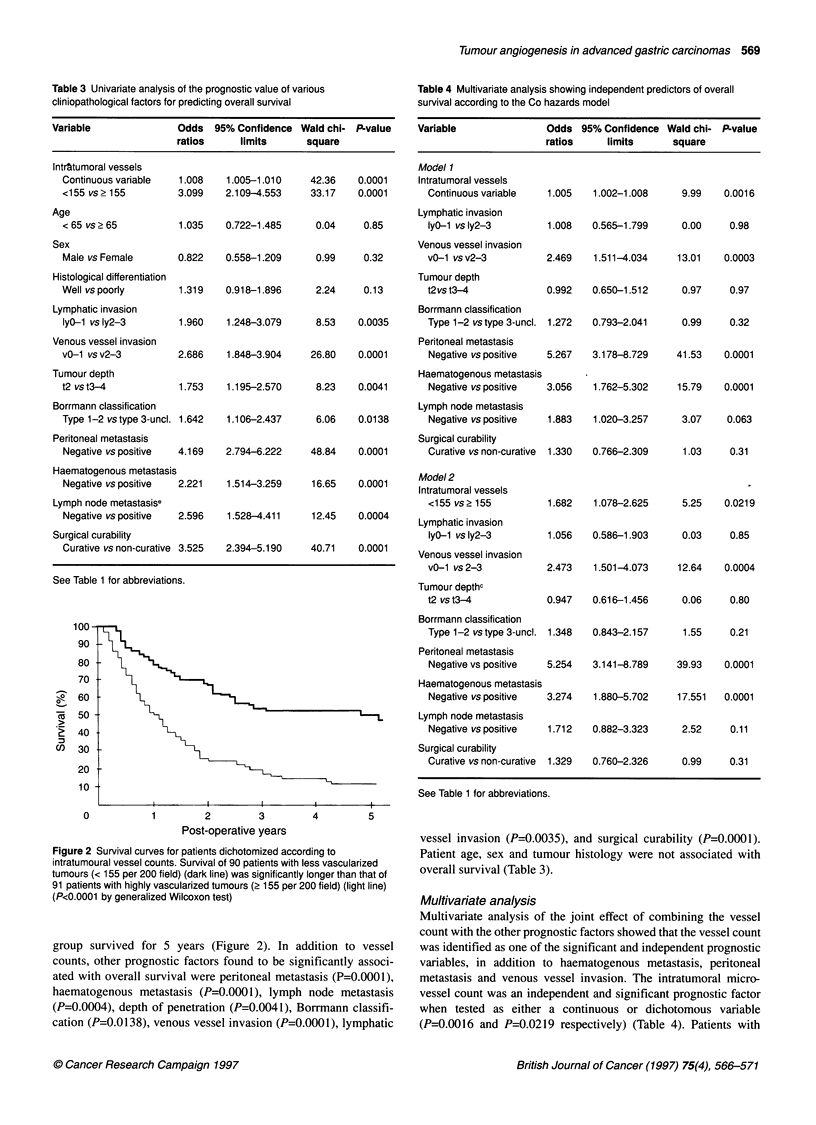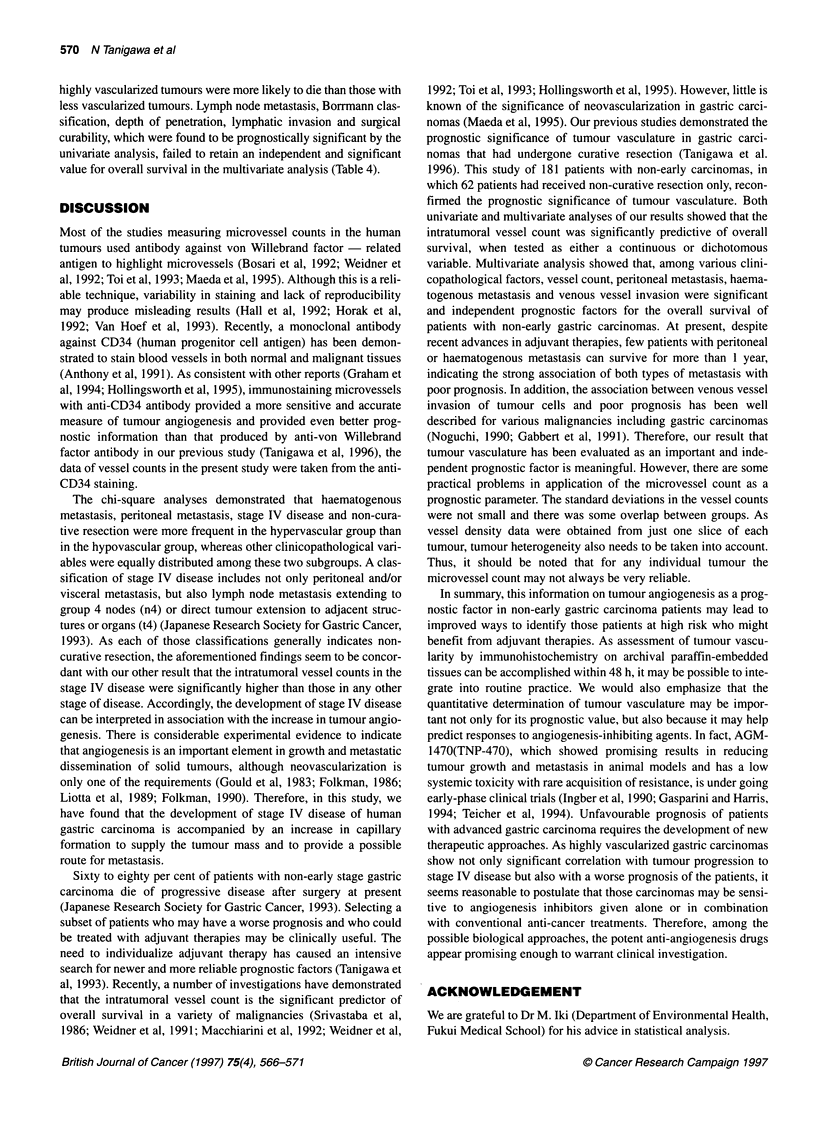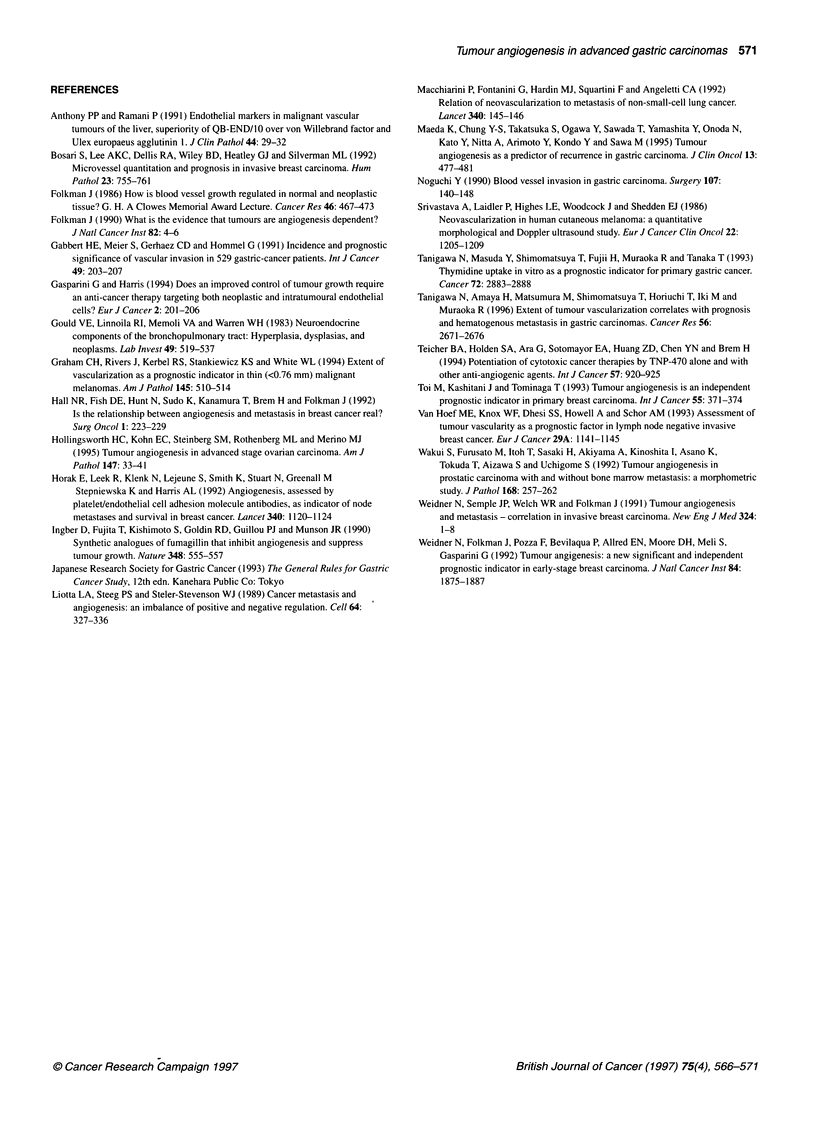# Association of tumour vasculature with tumour progression and overall survival of patients with non-early gastric carcinomas.

**DOI:** 10.1038/bjc.1997.99

**Published:** 1997

**Authors:** N. Tanigawa, H. Amaya, M. Matsumura, T. Shimomatsuya

**Affiliations:** Second Department of Surgery, Fukui Medical School, Japan.

## Abstract

In order to investigate the relationship between intratumoral vasculature and progression of gastric carcinomas and between vessel counts and survival of patients with non-early gastric carcinoma, we counted the intratumoral microvessels and compared their numbers with clinicopathological parameters, as well as with the patients' survival. Microvessels were stained with anti-CD34 monoclonal antibody before counting by microscopy (x200). In a group of 181 patients who had undergone tumour resection and were followed for more than 24 months the vessel counts for 83 patients with stage IV disease were significantly higher than those for patients with any other stage of disease. Among various clinicopathological variables, haematogenous metastasis, lymph node metastasis, peritoneal metastasis, stage IV disease and non-curative resection were more frequent in the patients with highly vascularized tumours (intratumoral vessel count > 155) than in those with less vascularized tumours. As a classification of stage IV disease such as haematogenous or peritoneal metastasis generally indicates non-curative resection, it can be considered that the development of stage IV disease is associated with the increase in tumour angiogenesis. Both univariate and multivariate analyses showed that the intratumoral vessel count was significantly predictive of overall survival, when tested as either a continuous or dichotomous variable. Cox hazards model analysis showed that the vessel count was one of the significant and independent prognostic variables. Patients with highly vascularized tumours were significantly more likely to die than those with less vascularized tumours. Assessment of tumour vasculature may therefore be important, not only for its prognostic value, but also as it may help to predict responses to angiogenesis-inhibiting agents.


British Journal of Cancer (1997) 75(4), 566–571
© 1997 Cancer Research Campaign

### Association of tumour vasculature with tumour progression and overall survival of patients with non-early gastric carcinomas

**N Tanigawa, H Amaya, M Matsumura and T Shimomatsuya**

The Second Department of Surgery, Fukui Medical School, Matsuoka-Cho, Yoshida-Gun, Fukui Prefecture 910–11, Japan

**Summary** In order to investigate the relationship between intratumoral vasculature and progression of gastric carcinomas and between vessel counts and survival of patients with non-early gastric carcinoma, we counted the intratumoral microvessels and compared their numbers with clinicopathological parameters, as well as with the patients' survival. Microvessels were stained with anti-CD34 monoclonal antibody before counting by microscopy (×200). In a group of 181 patients who had undergone tumour resection and were followed for more than 24 months the vessel counts for 83 patients with stage IV disease were significantly higher than those for patients with any other stage of disease. Among various clinicopathological variables, haematogenous metastasis, lymph node metastasis, peritoneal metastasis, stage IV disease and non-curative resection were more frequent in the patients with highly vascularized tumours (intratumoral vessel count > 155) than in those with less vascularized tumours. As a classification of stage IV disease such as haematogenous or peritoneal metastasis generally indicates non-curative resection, it can be considered that the development of stage IV disease is associated with the increase in tumour angiogenesis. Both univariate and multivariate analyses showed that the intratumoral vessel count was significantly predictive of overall survival, when tested as either a continuous or dichotomous variable. Cox hazards model analysis showed that the vessel count was one of the significant and independent prognostic variables. Patients with highly vascularized tumours were significantly more likely to die than those with less vascularized tumours. Assessment of tumour vasculature may therefore be important, not only for its prognostic value, but also as it may help to predict responses to angiogenesis-inhibiting agents.

**Keywords:** tumour vasculature; CD34; prognostic factor; gastric carcinoma

It is clinically valuable to identify those patients with gastric carcinoma at significant risk for recurrence who would benefit from treatment with adjuvant therapy. The need to individualize adjuvant therapy has resulted in an intensive search for newer and more reliable prognostic factors for gastric carcinomas. However, as far as we know, there is no parameter more suitable than the classification of disease stages to predict the patient's outcome. Whereas postoperative 5-year survival rates are 90–95% for early gastric carcinomas, which are confined to mucosal or submucosal layer (t1), only 20–40% of the patients with non-early stage carcinoma are expected to survive for 5 years or more (Japanese Research Society for Gastric Cancer, 1993). Recently, assessment of tumour vasculature has shown promise as a reliable prognostic marker in patients with a variety of malignancies, including malignant melanoma (Srivastava et al, 1986), breast carcinoma (Weidner et al, 1991; Toi et al, 1993), ovarian carcinoma (Hollingsworth et al, 1995), non-small-cell lung carcinoma (Macchiarini et al, 1992) and prostatic carcinoma (Wakui et al, 1992). We previously demonstrated that the intensity of the angiogenic response correlates with not only the overall survival rate, but also the development of metachronous haematogenous metastasis in the patients with gastric carcinomas who had undergone curative resection (Tanigawa et al, 1996). Therefore, in the present study, we attempted to clarify whether the extent of tumour vasculature would be prognostically important even when the study only included patients with non-early gastric carcinomas, some of whom had undergone non-curative resection.

*Received 4 April 1996*
*Revised 7 August 1996*
*Accepted 28 August 1996*

*Correspondence to:* N Tanigawa

### MATERIALS AND METHODS

### Patient characteristics

A total of 181 patients with a non-early stage of gastric carcinoma and who had undergone gastrectomy at our institution from October 1983 to December 1993 were studied. Preoperative endoscopic biopsies revealed that all the tumours were adenocarcinomas. Pathological diagnosis of each tumour was performed by pathologists in our hospital according to the General Rules for Gastric Cancer (Japanese Research Society for Gastric Cancer, 1993). Survivors were followed up for more than 2 years after surgery. There was no patient with other previous or concomitant primary cancer. The distribution of clinicopathological data for the entire population is listed in Table 1. Patients had received neither chemotherapy nor radiation therapy before surgery. A total of 107 patients (59%), less than 70 years old, were treated with tegaful (2-tetra-hydrofuryl)-5-fluorouracil at 600 mg $day^{-1}$ for as long as possible, up to 2 years after surgery. Each patient was examined at 2- or 3-monthly intervals in the outpatient clinic. The patients who failed to attend the clinic were traced by telephone. If the patient had died, the date and cause of death were recorded. Tumour specimens were fixed in 10% buffered formalin and embedded in paraffin. Histological grading was performed on haematoxylin and eosin (H&E)-stained sections.

### Highlighting endothelial cells

Immunohistochemical studies were performed with the method previously described (Tanigawa et al, 1996). Briefly, on formalin-fixed and paraffin-embedded tissue, the avidin–biotin immunoperoxidase complex technique was applied for highlighting the endothelial cells. One representative paraffin block from each case, in which a viable tumour was present, was used for this study. Sections (4–6 μm thick) mounted on glass slides were dewaxed in xylene, rehydrated in ethanol and then incubated with 3% hydrogen peroxide for 5 min. After washing with phosphate-buffered saline (PBS), they were incubated in 10% normal bovine serum for 5 min followed by incubation overnight with anti-CD34 monoclonal antibody (QB-END/10, Novocastra, Newcastle, UK) at a 1:25 dilution. A biotinylated goat anti-mouse immunoglobulin (Dako LSAB kit, Dako Japan, Kyoto, Japan) was used as a secondary antibody. Peroxidase-conjugated avidin (Dako Japan) was used at a dilution of 1:500. Finally, 0.02% diaminobenzidine and 1% hydrogen peroxide (Dako Japan) in PBS were used as the substrate. The sections were counterstained with haematoxylin.

### Assessment of tumour vasculature

Slides were examined under low power (40×) to identify the region of highest vessel density. For each slide, the five most vascular areas within the tumour mass were chosen. A 200× field in each of these five areas was counted, and the average counts of the five fields were recorded. A vessel lumen was not required for identification of a microvessel; single cells or cell clusters were counted. Large vessels with thick muscular walls or with lumina greater than 50 μm were excluded from the count. The microvessels were counted simultaneously by two investigators, MM and HA, who had no knowledge of the other prognostic factors and/or clinical outcomes, using a double-headed light microscope simultaneously.

### Statistical evaluation

We analysed the intratumoral vessel counts in two ways. First, we assessed vessel counts as a continuous variable and second as a dichotomous variable, which was dichotomized at a median number of vessel counts. The clinical characteristics of the patients in relation to the vessel counts were compared and checked by the chi-square test. Intratumoral vessel densities among various disease stages were compared and checked by Welch's t-test. The patterns of overall survival were estimated by means of the Kaplan–Meier method and their statistical differences were analysed by the generalized Wilcoxon test. The role of each of the prognostic variables (univariate analysis) and their joint effects (multivariate analysis) was evaluated. In the multivariate analysis, all variables with odds ratios significantly different from 1.0 in the univariate analysis were considered. For all statistical analyses, the Statistical Analysis System for personal computers (SAS Institute, Cary, NC, USA) was used, with significance having been defined as $P<0.05$.

### RESULTS

### Clinical outcome of all patients

Fifty-four of 181 patients (30%) have survived for a mean survival length of 49 months, ranging from 24 to 120 months. The remaining 127 (70%) died between 1 and 77 months (mean 17 months) after surgery. Peritoneal metastasis occurred in 79 patients (44%); 41 metastases were found at surgery and 38 developed after surgery. Haematogenous metastasis occurred in 38

**Table 1** Clinicopathological parameters in 181 non-early gastric carcinomas

| | |
|---|---|
| Age (years) | |
| Mean | 65 |
| Range | 26–87 |
| Sex | |
| Male | 121 (67%) |
| Female | 60 (33%) |
| Borrmann classification | |
| 1 | 14 (8%) |
| 2 | 52 (29%) |
| 3 | 78 (43%) |
| 4 | 27 (15%) |
| Uncl.[a] | 10 (6%) |
| Tumour histology[b] | |
| Pap | 19 (11%) |
| Tub | 44 (24%) |
| Poor | 69 (38%) |
| Undiff | 49 (27%) |
| Tumour depth[c] | |
| t2 | 72 (40%) |
| t3 | 79 (44%) |
| t4 | 30 (16%) |
| Lymphatic invasion[d] | |
| ly0 | 20 (11%) |
| ly1 | 28 (15%) |
| ly2 | 108 (60%) |
| ly3 | 25 (14%) |
| Vessel invasion[e] | |
| v0 | 59 (33%) |
| v1 | 56 (31%) |
| v2 | 58 (32%) |
| v3 | 8 (4%) |
| Lymph node metastasis[f] | |
| n0 | 36 (20%) |
| n1 | 46 (25%) |
| n2 | 48 (27%) |
| n3 | 20 (11%) |
| n4 | 31 (17%) |
| Stage of disease | |
| I | 22 (12%) |
| II | 20 (11%) |
| III | 56 (31%) |
| IV | 83 (46%) |
| Extent of gastrectomy | |
| Distal | 105 (58%) |
| Proximal | 8 (4%) |
| Total | 68 (38%) |
| Surgical curability | |
| Curative resection | 119 (66%) |
| Non-curative resection | 62 (34%) |

[a]Uncl.; unclassified type, being impossible to be classified as Borrmann 1 to 4, [b]Pap, papillary; Tub, tubular; Poor, poorly differentiated; Undiff, undifferentiated. [c]t2, invading the muscularis or the subserosa, t3, penetrating the serosa; t4, invading adjacent organs. [d]ly0, no lymphatic invasion; ly1, slight degree of lymphatic vessel invasion; ly2, moderate degree of lymphatic vessel invasion; ly3, extensive degree of lymphatic vessel invasion [e]v0, no venous vessel invasion; v1, slight degree of venous vessel invasion; v2, moderate degree of venous vessel invasion; v3, extensive degree of venous vessel invasion. [f]n0, no regional lymph node metastasis; n1, n2, n3, n4, metastasis in group 1, 2, 3, 4 lymph node stations respectively.

© Cancer Research Campaign 1997 

**Figure 1** Intratumoral vessel counts for each disease stage. Vessel counts for stage IV disease were significantly higher than those for any other stage of disease. *$P$=0.011, **$P$=0.003, ***$P$=0.011

patients (21%); 18 liver metastases were found at surgery and 20 haematogenous metastases developed after surgery (ten liver, four lung, two bone, two bone marrow and two brain). Both peritoneal and haematogenous metastases occurred in ten patients (6%); peritoneal and liver metastases were found in nine patients at surgery, and peritoneal and lung metastases developed after surgery in one patient. Lymph node metastases were found in 144 patients (80%) at surgery.

### Comparison of tumour vasculature among various clinicopathological factors

Intratumoral vessel counts for a total of 181 patients were 158 ± 75 mean plus or minus standard deviation (m.s.d.) (median 155). Each patient was assigned to one of the following stage groups according to the clinicopathological findings of the resected tumours: 22 patients (12%) with stage I disease, 20 (11%) with stage II, 56 (31%) with stage III and 83 (46%) with stage IV. Intratumoral vessel counts for relevant disease stages were 143 ± 65 MSD for stage I disease, 124 ± 69 MSD for stage II, 144 ± 84 MSD for stage III, and 179 ± 69 MSD for stage IV. Vessel counts for stage IV disease were significantly higher than those for any other stage of disease (Figure 1). A median value of 155 was taken as the cut-off point for discrimination of the 181 patients into two subgroups: 91 patients with hypovascular tumours and 90 with hypervascular tumours. Among the clinicopathological variables examined, age, sex, Borrmann classification, tumour histology, depth of penetration, lymphatic invasion, venous vessel invasion and adjuvant chemotherapy were equally distributed among these two subgroups. However, haematogenous metastasis, lymph node metastasis, peritoneal metastasis, stage IV disease and non-curative resection were more frequent in the hypervascular group (Table 2).

**Table 2** Comparison of clinicopathological features among patients with hypovascular tumours and those with hypervascular tumours

| Variable | Hypovascular ($n$=90) (%) | Hypervascular ($n$=91) (%) | $P$-value |
|---|---|---|---|
| Age | | | |
| ≤65 | 45 (52) | 41 (48) | NS |
| >65 | 45 (47) | 50 (53) | |
| Sex | | | |
| Male | 57 (47) | 64 (53) | NS |
| Female | 33 (55) | 27 (45) | |
| Borrmann classification | | | |
| 1, 2 | 32 (50) | 32 (50) | NS |
| 3, 4, unclassified[a] | 58 (50) | 59 (50) | |
| Tumour histology | | | |
| Well differentiated | 47 (57) | 36 (43) | NS |
| Poorly differentiated | 43 (44) | 55 (56) | |
| Depth of penetration[b] | | | |
| t2 | 41 (56) | 32 (44) | NS |
| t3, t4 | 49 (45) | 59 (55) | |
| Lymphatic invasion[c] | | | |
| ly0, ly1 | 25 (51) | 24 (49) | NS |
| ly2, ly3 | 65 (49) | 67 (51) | |
| Venous vessel invasion[d] | | | |
| v0, v1 | 64 (54) | 54 (46) | NS |
| v2, v3 | 26 (41) | 37 (59) | |
| Adjuvant chemotherapy | | | |
| Negative | 55 (51) | 52 (49) | NS |
| Positive | 35 (47) | 39 (53) | |
| Peritoneal metastasis | | | |
| Negative | 53 (58) | 39 (42) | $P$=0.031 |
| Positive | 37 (41) | 52 (59) | |
| Lymph node metastasis[e] | | | |
| n0, n1, n2 | 69 (53) | 12 (47) | NS |
| n3, n4 | 21 (41) | 30 (59) | |
| Haematogenous metastasis | | | |
| Negative | 81 (61) | 52 (39) | $P$<0.000001 |
| Positive | 9 (19) | 39 (81) | |
| Stage of disease | | | |
| Stage I | 14 (64) | 8 (36) | $P$=0.001 |
| Stage II | 14 (70) | 6 (30) | |
| Stage III | 34 (61) | 22 (39) | |
| Stage IV | 28 (34) | 55 (66) | |
| Surgical curability | | | |
| Curative | 69 (58) | 50 (42) | $P$=0.002 |
| Non-curative | 21 (34) | 41 (66) | |

See Table 1 for abbreviations.

### Correlation between vessel counts and overall survival

### *Univariate analysis*

We found that the intratumoral vessel counts significantly predicted overall survival when evaluated as either a continuous or dichotomous variable ($P$=0.0001 and $P$=0.0001 respectively) (Table 3). The odds of death increase with the intratumoral microvessel counts and an average vessel count of less than 155 per 200x field suggested a better survival. The survival rates of the two subgroups were calculated as follows using the Kaplan–Meier method: 50% ± 6% standard error of the mean (s.e.m.) of the hypovascular group, but only 11% ± 4% s.e.m. of the hypervascular

© Cancer Research Campaign 1997

**Table 3** Univariate analysis of the prognostic value of various cliniopathological factors for predicting overall survival

| Variable | Odds ratios | 95% Confidence limits | Wald chi-square | *P*-value |
|---|---|---|---|---|
| Inträtumoral vessels | | | | |
| Continuous variable | 1.008 | 1.005–1.010 | 42.36 | 0.0001 |
| <155 *vs* ≥ 155 | 3.099 | 2.109–4.553 | 33.17 | 0.0001 |
| Age | | | | |
| < 65 *vs* ≥ 65 | 1.035 | 0.722–1.485 | 0.04 | 0.85 |
| Sex | | | | |
| Male *vs* Female | 0.822 | 0.558–1.209 | 0.99 | 0.32 |
| Histological differentiation | | | | |
| Well *vs* poorly | 1.319 | 0.918–1.896 | 2.24 | 0.13 |
| Lymphatic invasion | | | | |
| ly0–1 *vs* ly2–3 | 1.960 | 1.248–3.079 | 8.53 | 0.0035 |
| Venous vessel invasion | | | | |
| v0–1 *vs* v2–3 | 2.686 | 1.848–3.904 | 26.80 | 0.0001 |
| Tumour depth | | | | |
| t2 *vs* t3–4 | 1.753 | 1.195–2.570 | 8.23 | 0.0041 |
| Borrmann classification | | | | |
| Type 1–2 *vs* type 3-uncl. | 1.642 | 1.106–2.437 | 6.06 | 0.0138 |
| Peritoneal metastasis | | | | |
| Negative *vs* positive | 4.169 | 2.794–6.222 | 48.84 | 0.0001 |
| Haematogenous metastasis | | | | |
| Negative *vs* positive | 2.221 | 1.514–3.259 | 16.65 | 0.0001 |
| Lymph node metastasis[e] | | | | |
| Negative *vs* positive | 2.596 | 1.528–4.411 | 12.45 | 0.0004 |
| Surgical curability | | | | |
| Curative *vs* non-curative | 3.525 | 2.394–5.190 | 40.71 | 0.0001 |

See Table 1 for abbreviations.

**Figure 2** Survival curves for patients dichotomized according to intratumoural vessel counts. Survival of 90 patients with less vascularized tumours (< 155 per 200 field) (dark line) was significantly longer than that of 91 patients with highly vascularized tumours (≥ 155 per 200 field) (light line) (*P*<0.0001 by generalized Wilcoxon test)

group survived for 5 years (Figure 2). In addition to vessel counts, other prognostic factors found to be significantly associated with overall survival were peritoneal metastasis (P=0.0001), haematogenous metastasis (*P*=0.0001), lymph node metastasis (*P*=0.0004), depth of penetration (*P*=0.0041), Borrmann classification (*P*=0.0138), venous vessel invasion (*P*=0.0001), lymphatic vessel invasion (*P*=0.0035), and surgical curability (*P*=0.0001). Patient age, sex and tumour histology were not associated with overall survival (Table 3).

**Table 4** Multivariate analysis showing independent predictors of overall survival according to the Co hazards model

| Variable | Odds ratios | 95% Confidence limits | Wald chi-square | *P*-value |
|---|---|---|---|---|
| *Model 1* | | | | |
| Intratumoral vessels | | | | |
| Continuous variable | 1.005 | 1.002–1.008 | 9.99 | 0.0016 |
| Lymphatic invasion | | | | |
| ly0–1 *vs* ly2–3 | 1.008 | 0.565–1.799 | 0.00 | 0.98 |
| Venous vessel invasion | | | | |
| v0–1 *vs* v2–3 | 2.469 | 1.511–4.034 | 13.01 | 0.0003 |
| Tumour depth | | | | |
| t2 *vs* t3–4 | 0.992 | 0.650–1.512 | 0.97 | 0.97 |
| Borrmann classification | | | | |
| Type 1–2 *vs* type 3-uncl. | 1.272 | 0.793–2.041 | 0.99 | 0.32 |
| Peritoneal metastasis | | | | |
| Negative *vs* positive | 5.267 | 3.178–8.729 | 41.53 | 0.0001 |
| Haematogenous metastasis | | | | |
| Negative *vs* positive | 3.056 | 1.762–5.302 | 15.79 | 0.0001 |
| Lymph node metastasis | | | | |
| Negative *vs* positive | 1.883 | 1.020–3.257 | 3.07 | 0.063 |
| Surgical curability | | | | |
| Curative *vs* non-curative | 1.330 | 0.766–2.309 | 1.03 | 0.31 |
| *Model 2* | | | | |
| Intratumoral vessels | | | | |
| <155 *vs* ≥ 155 | 1.682 | 1.078–2.625 | 5.25 | 0.0219 |
| Lymphatic invasion | | | | |
| ly0–1 *vs* ly2–3 | 1.056 | 0.586–1.903 | 0.03 | 0.85 |
| Venous vessel invasion | | | | |
| v0–1 *vs* 2–3 | 2.473 | 1.501–4.073 | 12.64 | 0.0004 |
| Tumour depth[c] | | | | |
| t2 *vs* t3–4 | 0.947 | 0.616–1.456 | 0.06 | 0.80 |
| Borrmann classification | | | | |
| Type 1–2 *vs* type 3-uncl. | 1.348 | 0.843–2.157 | 1.55 | 0.21 |
| Peritoneal metastasis | | | | |
| Negative vs positive | 5.254 | 3.141–8.789 | 39.93 | 0.0001 |
| Haematogenous metastasis | | | | |
| Negative *vs* positive | 3.274 | 1.880–5.702 | 17.551 | 0.0001 |
| Lymph node metastasis | | | | |
| Negative *vs* positive | 1.712 | 0.882–3.323 | 2.52 | 0.11 |
| Surgical curability | | | | |
| Curative *vs* non-curative | 1.329 | 0.760–2.326 | 0.99 | 0.31 |

See Table 1 for abbreviations.

### *Multivariate analysis*

Multivariate analysis of the joint effect of combining the vessel count with the other prognostic factors showed that the vessel count was identified as one of the significant and independent prognostic variables, in addition to haematogenous metastasis, peritoneal metastasis and venous vessel invasion. The intratumoral microvessel count was an independent and significant prognostic factor when tested as either a continuous or dichotomous variable (*P*=0.0016 and *P*=0.0219 respectively) (Table 4). Patients with highly vascularized tumours were more likely to die than those with less vascularized tumours. Lymph node metastasis, Borrmann classification, depth of penetration, lymphatic invasion and surgical curability, which were found to be prognostically significant by the univariate analysis, failed to retain an independent and significant value for overall survival in the multivariate analysis (Table 4).

### DISCUSSION

Most of the studies measuring microvessel counts in the human tumours used antibody against von Willebrand factor — related antigen to highlight microvessels (Bosari et al, 1992; Weidner et al, 1992; Toi et al, 1993; Maeda et al, 1995). Although this is a reliable technique, variability in staining and lack of reproducibility may produce misleading results (Hall et al, 1992; Horak et al, 1992; Van Hoef et al, 1993). Recently, a monoclonal antibody against CD34 (human progenitor cell antigen) has been demonstrated to stain blood vessels in both normal and malignant tissues (Anthony et al, 1991). As consistent with other reports (Graham et al, 1994; Hollingsworth et al, 1995), immunostaining microvessels with anti-CD34 antibody provided a more sensitive and accurate measure of tumour angiogenesis and provided even better prognostic information than that produced by anti-von Willebrand factor antibody in our previous study (Tanigawa et al, 1996), the data of vessel counts in the present study were taken from the anti-CD34 staining.

The chi-square analyses demonstrated that haematogenous metastasis, peritoneal metastasis, stage IV disease and non-curative resection were more frequent in the hypervascular group than in the hypovascular group, whereas other clinicopathological variables were equally distributed among these two subgroups. A classification of stage IV disease includes not only peritoneal and/or visceral metastasis, but also lymph node metastasis extending to group 4 nodes (n4) or direct tumour extension to adjacent structures or organs (t4) (Japanese Research Society for Gastric Cancer, 1993). As each of those classifications generally indicates non-curative resection, the aforementioned findings seem to be concordant with our other result that the intratumoral vessel counts in the stage IV disease were significantly higher than those in any other stage of disease. Accordingly, the development of stage IV disease can be interpreted in association with the increase in tumour angiogenesis. There is considerable experimental evidence to indicate that angiogenesis is an important element in growth and metastatic dissemination of solid tumours, although neovascularization is only one of the requirements (Gould et al, 1983; Folkman, 1986; Liotta et al, 1989; Folkman, 1990). Therefore, in this study, we have found that the development of stage IV disease of human gastric carcinoma is accompanied by an increase in capillary formation to supply the tumour mass and to provide a possible route for metastasis.

Sixty to eighty per cent of patients with non-early stage gastric carcinoma die of progressive disease after surgery at present (Japanese Research Society for Gastric Cancer, 1993). Selecting a subset of patients who may have a worse prognosis and who could be treated with adjuvant therapies may be clinically useful. The need to individualize adjuvant therapy has caused an intensive search for newer and more reliable prognostic factors (Tanigawa et al, 1993). Recently, a number of investigations have demonstrated that the intratumoral vessel count is the significant predictor of overall survival in a variety of malignancies (Srivastaba et al, 1986; Weidner et al, 1991; Macchiarini et al, 1992; Weidner et al, 1992; Toi et al, 1993; Hollingsworth et al, 1995). However, little is known of the significance of neovascularization in gastric carcinomas (Maeda et al, 1995). Our previous studies demonstrated the prognostic significance of tumour vasculature in gastric carcinomas that had undergone curative resection (Tanigawa et al. 1996). This study of 181 patients with non-early carcinomas, in which 62 patients had received non-curative resection only, reconfirmed the prognostic significance of tumour vasculature. Both univariate and multivariate analyses of our results showed that the intratumoral vessel count was significantly predictive of overall survival, when tested as either a continuous or dichotomous variable. Multivariate analysis showed that, among various clinicopathological factors, vessel count, peritoneal metastasis, haematogenous metastasis and venous vessel invasion were significant and independent prognostic factors for the overall survival of patients with non-early gastric carcinomas. At present, despite recent advances in adjuvant therapies, few patients with peritoneal or haematogenous metastasis can survive for more than 1 year, indicating the strong association of both types of metastasis with poor prognosis. In addition, the association between venous vessel invasion of tumour cells and poor prognosis has been well described for various malignancies including gastric carcinomas (Noguchi, 1990; Gabbert et al, 1991). Therefore, our result that tumour vasculature has been evaluated as an important and independent prognostic factor is meaningful. However, there are some practical problems in application of the microvessel count as a prognostic parameter. The standard deviations in the vessel counts were not small and there was some overlap between groups. As vessel density data were obtained from just one slice of each tumour, tumour heterogeneity also needs to be taken into account. Thus, it should be noted that for any individual tumour the microvessel count may not always be very reliable.

In summary, this information on tumour angiogenesis as a prognostic factor in non-early gastric carcinoma patients may lead to improved ways to identify those patients at high risk who might benefit from adjuvant therapies. As assessment of tumour vascularity by immunohistochemistry on archival paraffin-embedded tissues can be accomplished within 48 h, it may be possible to integrate into routine practice. We would also emphasize that the quantitative determination of tumour vasculature may be important not only for its prognostic value, but also because it may help predict responses to angiogenesis-inhibiting agents. In fact, AGM-1470(TNP-470), which showed promising results in reducing tumour growth and metastasis in animal models and has a low systemic toxicity with rare acquisition of resistance, is under going early-phase clinical trials (Ingber et al, 1990; Gasparini and Harris, 1994; Teicher et al, 1994). Unfavourable prognosis of patients with advanced gastric carcinoma requires the development of new therapeutic approaches. As highly vascularized gastric carcinomas show not only significant correlation with tumour progression to stage IV disease but also with a worse prognosis of the patients, it seems reasonable to postulate that those carcinomas may be sensitive to angiogenesis inhibitors given alone or in combination with conventional anti-cancer treatments. Therefore, among the possible biological approaches, the potent anti-angiogenesis drugs appear promising enough to warrant clinical investigation.

### ACKNOWLEDGEMENT

We are grateful to Dr M. Iki (Department of Environmental Health, Fukui Medical School) for his advice in statistical analysis.

© Cancer Research Campaign 1997

### REFERENCES

Anthony PP and Ramani P (1991) Endothelial markers in malignant vascular tumours of the liver, superiority of QB-END/10 over von Willebrand factor and Ulex europaeus agglutinin 1. *J Clin Pathol* **44**: 29–32

Bosari S, Lee AKC, Dellis RA, Wiley BD, Heatley GJ and Silverman ML (1992) Microvessel quantitation and prognosis in invasive breast carcinoma. *Hum Pathol* **23**: 755–761

Folkman J (1986) How is blood vessel growth regulated in normal and neoplastic tissue? G. H. A Clowes Memorial Award Lecture. *Cancer Res* **46**: 467–473

Folkman J (1990) What is the evidence that tumours are angiogenesis dependent? *J Natl Cancer Inst* **82**: 4–6

Gabbert HE, Meier S, Gerhaez CD and Hommel G (1991) Incidence and prognostic significance of vascular invasion in 529 gastric-cancer patients. *Int J Cancer* **49**: 203–207

Gasparini G and Harris (1994) Does an improved control of tumour growth require an anti-cancer therapy targeting both neoplastic and intratumoural endothelial cells? *Eur J Cancer* **2**: 201–206

Gould VE, Linnoila RI, Memoli VA and Warren WH (1983) Neuroendocrine components of the bronchopulmonary tract: Hyperplasia, dysplasias, and neoplasms. *Lab Invest* **49**: 519–537

Graham CH, Rivers J, Kerbel RS, Stankiewicz KS and White WL (1994) Extent of vascularization as a prognostic indicator in thin (<0.76 mm) malignant melanomas. *Am J Pathol* **145**: 510–514

Hall NR, Fish DE, Hunt N, Sudo K, Kanamura T, Brem H and Folkman J (1992) Is the relationship between angiogenesis and metastasis in breast cancer real? *Surg Oncol* **1**: 223–229

Hollingsworth HC, Kohn EC, Steinberg SM, Rothenberg ML and Merino MJ (1995) Tumour angiogenesis in advanced stage ovarian carcinoma. *Am J Pathol* **147**: 33–41

Horak E, Leek R, Klenk N, Lejeune S, Smith K, Stuart N, Greenall M Stepniewska K and Harris AL (1992) Angiogenesis, assessed by platelet/endothelial cell adhesion molecule antibodies, as indicator of node metastases and survival in breast cancer. *Lancet* **340**: 1120–1124

Ingber D, Fujita T, Kishimoto S, Goldin RD, Guillou PJ and Munson JR (1990) Synthetic analogues of fumagillin that inhibit angiogenesis and suppress tumour growth. *Nature* **348**: 555–557

Japanese Research Society for Gastric Cancer (1993) *The General Rules for Gastric Cancer Study*, 12th edn. Kanehara Public Co: Tokyo

Liotta LA, Steeg PS and Steler-Stevenson WJ (1989) Cancer metastasis and angiogenesis: an imbalance of positive and negative regulation. *Cell* **64**: 327–336

Macchiarini P, Fontanini G, Hardin MJ, Squartini F and Angeletti CA (1992) Relation of neovascularization to metastasis of non-small-cell lung cancer. *Lancet* **340**: 145–146

Maeda K, Chung Y-S, Takatsuka S, Ogawa Y, Sawada T, Yamashita Y, Onoda N, Kato Y, Nitta A, Arimoto Y, Kondo Y and Sawa M (1995) Tumour angiogenesis as a predictor of recurrence in gastric carcinoma. *J Clin Oncol* **13**: 477–481

Noguchi Y (1990) Blood vessel invasion in gastric carcinoma. *Surgery* **107**: 140–148

Srivastava A, Laidler P, Highes LE, Woodcock J and Shedden EJ (1986) Neovascularization in human cutaneous melanoma: a quantitative morphological and Doppler ultrasound study. *Eur J Cancer Clin Oncol* **22**: 1205–1209

Tanigawa N, Masuda Y, Shimomatsuya T, Fujii H, Muraoka R and Tanaka T (1993) Thymidine uptake in vitro as a prognostic indicator for primary gastric cancer. *Cancer* **72**: 2883–2888

Tanigawa N, Amaya H, Matsumura M, Shimomatsuya T, Horiuchi T, Iki M and Muraoka R (1996) Extent of tumour vascularization correlates with prognosis and hematogenous metastasis in gastric carcinomas. *Cancer Res* **56**: 2671–2676

Teicher BA, Holden SA, Ara G, Sotomayor EA, Huang ZD, Chen YN and Brem H (1994) Potentiation of cytotoxic cancer therapies by TNP-470 alone and with other anti-angiogenic agents. *Int J Cancer* **57**: 920–925

Toi M, Kashitani J and Tominaga T (1993) Tumour angiogenesis is an independent prognostic indicator in primary breast carcinoma. *Int J Cancer* **55**: 371–374

Van Hoef ME, Knox WF, Dhesi SS, Howell A and Schor AM (1993) Assessment of tumour vascularity as a prognostic factor in lymph node negative invasive breast cancer. *Eur J Cancer* **29A**: 1141–1145

Wakui S, Furusato M, Itoh T, Sasaki H, Akiyama A, Kinoshita I, Asano K, Tokuda T, Aizawa S and Uchigome S (1992) Tumour angiogenesis in prostatic carcinoma with and without bone marrow metastasis: a morphometric study. *J Pathol* **168**: 257–262

Weidner N, Semple JP, Welch WR and Folkman J (1991) Tumour angiogenesis and metastasis – correlation in invasive breast carcinoma. *New Eng J Med* **324**: 1–8

Weidner N, Folkman J, Pozza F, Bevilaqua P, Allred EN, Moore DH, Meli S, Gasparini G (1992) Tumour angigenesis: a new significant and independent prognostic indicator in early-stage breast carcinoma. *J Natl Cancer Inst* **84**: 1875–1887

© Cancer Research Campaign 1997